# ST Elevation: Telling Pathology from the Benign Patterns

**DOI:** 10.5539/gjhs.v4n3p51

**Published:** 2012-05-01

**Authors:** Waleed Tallat Kayani, Henry Darchon Huang, Salman Bandeali, James M. Wilson, Salim Virani, Yochai Birnbaum

**Affiliations:** 1 Department of Medicine, Baylor College of Medicine, One Baylor Plaza-Faculty Center, Houston, Texas, USA; 2 Department of Cardiology, Texas Heart Institute, St. Luke’s Episcopal Hospital, Houston, Texas, USA; 3 Health Policy and Quality Program, Michael E. DeBakey Veteran Affairs Medical Center Health Services Research and Development Center of Excellence. Houston, Texas; 4 Section of Cardiology, Michael E. DeBakey Veterans Affairs Medical Center, Houston. Texas, USA

**Keywords:** ST elevation, non ischemic ST elevation

## Abstract

Benefits of early reperfusion in patients presenting with acute ST elevation myocardial infarction (STEMI) are well known. The American College of Cardiology / American Heart Association guidelines recommend triage decisions are made within 10 minutes of performing initial electrocardiogram (ECG). Since many patients presenting with ischemic symptoms may have ST elevation (STE) at baseline, not all STE signify transmural ischemia. Benign patterns can be easy to find in some cases. However, patients with benign STE at baseline (left ventricular hypertrophy, early repolarization pattern) may have ongoing ischemia and present with Non-ST elevation myocardial infarction (NSTEMI) or even STEMI superimposed on the benign pattern. The ability of clinicians to distinguish between ischemic and non ischemic STE varies widely and is affected by prevalence of such changes in patient population. More studies need to be done to delineate the criteria to clearly distinguish between ischemic and non ischemic ST elevation.

## 1. Introduction

The electrocardiogram (ECG) plays an important part in appropriate management of patients presenting with chest pain. Patients diagnosed with acute STEMI are triaged to receive urgent reperfusion therapy with either primary percutaneous coronary intervention (pPCI) or thrombolytic therapy and those judged not to have ST elevation receive conservative treatment early on (Antman et al., 2004). The American College of Cardiology/American Heart Association (ACC/AHA) guidelines for STEMI recommend immediate reperfusion therapy in patients presenting within 12 hours of the onset of symptoms compatible with myocardial infarction (even if resolved) and concomitant STE in 2 or more adjacent leads (> 0.1 mV at J point) (Antman, et al., 2004). Thygesen et al, in their expert consensus document recommend different cutoffs for men and women in leads V2- V3 (Thygesen et al., 2007). They recommend STE at J point of ≥ 0.2 mV in men and ≥ 0.15 mV in women in leads V2- V3 and/or ≥ 0.1 mV in all other leads. Different cutoffs in interpretation of STE create ambiguity in the mind of ECG interpreters. A reasonable assumption would be that if criteria by Thygesen et al are used, reported prevalence of STEMI would be lower. Another factor needing consideration is that a significant proportion of patients with chest pain may have nonischemic ST elevation (NISTE) (Birnbaum, 2007; Brady, Perron, Martin, Beagle, & Aufderheide, 2001; Otto & Aufderheide, 1994; Wang, Asinger, & Marriott, 2003). The situation is further compounded by atypical presentations of myocardial infarction in diabetics, elderly patients and women (Canto et al., 2000; Deedwania & Carbajal, 1991; Kyker & Limacher, 2002). Although measuring of serum troponin levels may help in risk stratifying those with Non-ST elevation Acute Coronary Syndrome (NSTE-ACS), they cannot be utilized in diagnosing STEMI as a rise may not be seen for up to 6 hours after the onset of ischemia/infarction which would be too late for reperfusion decisions. Also, patients with baseline NISTE may present with NSTEMI and positive cardiac markers, therefore presence of positive cardiac markers is not an indication for acute reperfusion therapy by itself.

Reperfusion decisions should be made within 10 minutes of initial ECG in patients presenting with suspected STEMI according to ACC/AHA STEMI guidelines (Antman et al., 2008). Therefore, prompt and correct interpretation of ECG is of utmost importance (Zimetbaum & Josephson, 2003). NISTE is frequently encountered in practice. Some etiologies of NISTE can be easily differentiated from STEMI on an ECG tracing. It must be kept in mind, however, that some patients with prior NISTE (early repolarization or left ventricular hypertrophy) may also present with STEMI or NSTEMI. Therefore, a benign pattern of STE at baseline cannot be used to exclude the possibility of acute coronary syndrome. A subset of patients may have rare etiologies of NISTE which can be hard to identify with just an ECG in absence of accompanying clinical data. Rapid interpretation of ischemic changes becomes more challenging in such cases. Numerous etiologies can lead to STE and range from baseline STE without ischemia, to concomitant ischemia outside of coronary artery teritory (aortic dissection) and even conditions that present with chest pain but are non ischemic in nature (e.g. pericarditis or myocarditis). Given the current focus on reducing door-to-balloon time, there is a higher chance of over-diagnosing “STEMI” and thus subsequently exposing patients to unnecessary invasive procedures or potentially harmful medical therapies.

**Figure 1:**
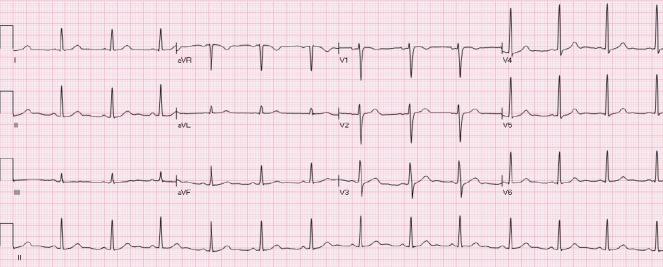
Normal ECG. No abnormal Q waves. ST is isoelectric in all leads

**Figure 2:**
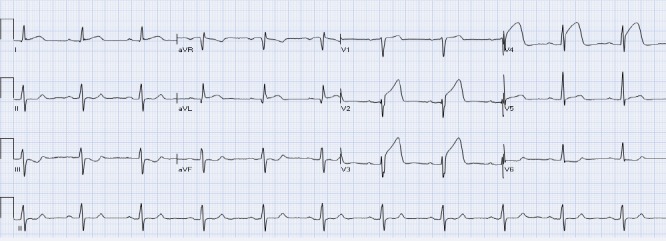
Acute anterior STEMI. There is ST elevation in leads aVL, V2-V4 with reciprocal ST depression in leads III and aVF

The ST segment is at the same horizontal level as that of adjacent T- P or P- R segments or iso-electric in the majority of individuals (Figure 1) and deviation of ST segment (elevation or depression) is usually considered a sign of ischemia. Figure 2 is an example of ST elevation due to acute anterior STEMI (caused by proximal occlusion of the left anterior descending coronary artery). Prevalence rates of NISTE as high as 15% have been reported in the general population. In a cohort of 6,014 US Air Force recruits aged between 16 and 58 years, Hiss et al reported a 91% prevalence of STE of 0.1 to 0.3 mV in more than 1 precordial leads- mostly lead V2 (Hiss, Lamb, & Allen, 1960). Similar high prevalence of NISTE in young males was reported by Surawicz et al (Surawicz & Parikh, 2002). They found a prevalence of STE >0.1 mV in 1 or more leads from V1-V4 in 93% of males aged 17 – 24 years, with a decrease to 30% in age > 76 years. As is evident by data mentioned, majority of men have STE > 0.1mV at baseline, also known as the male pattern which is considered normal. Surawicz et al reported STE of > 0.1 mV in 20% of women, which was not affected by age (Surawicz & Parikh, 2002). It needs to be mentioned however, that although Thygesen et al take this into consideration in their article expert consensus document, the ACC/AHA guidelines do not. A prospective study performed on 1345 consecutive patients to study rates of false activation of catheterization laboratory showed no clear culprit lesions in 14% of patients who underwent emergent cardiac catheterization for a suspected STEMI (Larson et al., 2007). Similar findings were observed in a recent Danish study as well (Sejersten et al., 2008). Common causes of NISTE are outlined in table 1. The purpose of this review is to discuss common non- ischemic causes of STE and present some methods to increase yield of interpretation that have been implemented with success in some clinical settings.

**Table 1:** Common causes of Non ischemic ST segment elevation (NISTE)

Early repolarization pattern Electrolyte abnormalities (hyperkalemia, hypercalcemia) Normal variant of STE (mainly leads V2- V3) STE secondary to left ventricular hypertrophy Pericarditis Intraventricular conduction delay Brugada’s syndrome Left ventricular aneurysm Takotsubo’s cardiomyopathy (apical ballooning syndrome) Wolf- Parkinson- White syndrome

STE- ST elevation

## 2. Common Causes of Non-Ischemic ST Elevation (NISTE)

### 2.1 “Concave” versus “Convex” Pattern of STE

Normally, the ST segment is iso-electric with the TR and TP segments and reflects ventricular depolarization. It is a classic teaching that STE with upwardly convex or straight is consistent with STEMI and STE which is concave is morphology is consistent with NISTE. The ACC/AHA guidelines also mention that STEMI is less likely if associated ST elevations are concave versus convex (Antman, et al., 2004). Difficulty in interpreting the shape of upslope of ST segment may be encountered by prominent T waves as they are concave in morphology and transition point between ST segment and beginning of T waves is not always obvious. However, concave ST segments cannot be relied upon to rule out ischemia as NISTE as a prevalence of 43% of concave STE in proven LAD territory infarctions has been reported in a study (Smith, 2006).

### 2.2 Early Repolarization

**Figure 3:**
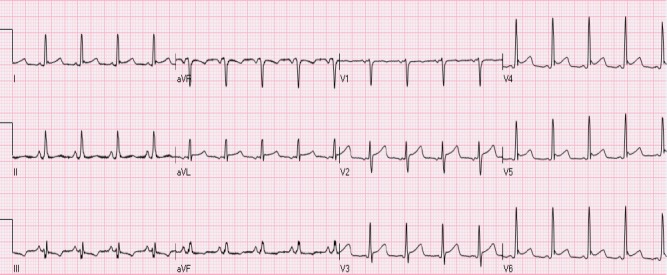
Early repolarization. There is mild STE with notched J-point in leads I, aVL, V4-V6. In addition, there is also mild STE in leads V2-V3, compatible with a “normal variant” (see below)

Early repolarization usually manifests as STE of 0.1- 0.4 mV in the lateral leads (mainly V5- V6), accompanied with a characteristic notch at J point (Figure 3). Involvement of inferior leads may also be observed. ST segment is usually concave with accompanying peaked, tall T waves. Early repolarization pattern is usually seen in young males. Interestingly, STE may be transient in many cases and decreases in amplitude or even resolves with hyperventilation and tachycardia. Hence, dynamic nature of ST segment is not always a reliable indicator of ischemia. Occasionally, T wave inversions may also be observed in precordial leads which are believed to be associated with juvenile T waves and should not be confused with ischemia. Comparison with previous ECGs can serve as a valuable tool. The underlying pathophysiology associated with early repolarization still remains a matter of controversy as some investigators consider it not to be secondary to early repolarization of the ventricle (Mirvis, 1982). Early repolarization NISTE was previously thought to be benign in etiology given nearly 5% prevalence in normal population (Klatsky, Oehm, Cooper, Udaltsova, & Armstrong, 2003; Tikkanen et al., 2009). However, there is recent data to suggest its association with fatal arrhythmias, especially if there is STE of > 0.2 mV (Haissaguerre et al., 2008; Myerburg & Castellanos, 2008; Nam, Kim, & Antzelevitch, 2008; Tikkanen, et al., 2009).

Prominent J point notching- also known as Osborne waves may be seen transiently in hypothermia (Spodick, 2006), and should not be confused with benign early repolarization. Bradycardia and tremor are frequently seen with hypothermia. Osborne waves may also be seen in hypercalcemia and nervous system disorders. QT interval prolongation is seen with hypothermia whereas hypercalcemia is usually associated with QT interval shortening (Nishi, Barbagelata, Atar, Birnbaum, & Tuero, 2006). Hyperkalemia is another cause of ST elevation but is accompanied by widening of QRS interval and changes in PR segment. Another etiology that can be confused with J point notching is epsilon waves (observed only in leads V1- V3) in Arrhythmogenic right ventricular dysplasia. Their limited distribution in leads V1- V3 serves as a distinguishing feature (Nasir et al., 2004).

### 2.3 The “Normal-Variant” Pattern

**Figure 4:**
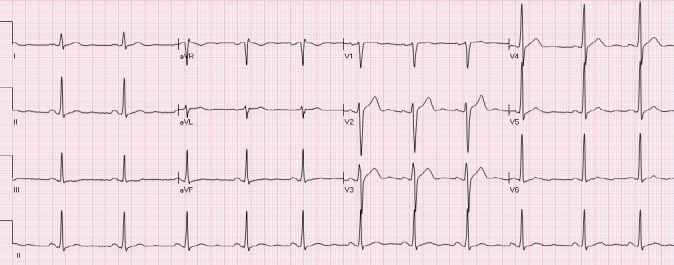
A patient with a “normal variant” STE in leads V2-V3. The ST is concave and T waves are positive

Leads V1- V3 most commonly demonstrate the normal variant pattern (Wang, et al., 2003) (Figure 4). It is observed more frequently in young African American and Hispanic males. Lack of QRS voltage criteria for left ventricular hypertrophy (LVH) and absence of concomitant ST depressions in lateral leads differentiates it from NISTE associated with LVH. “Normal variant” and “early repolarization” patterns are considered to be the same by some investigators. The patterns do occur simultaneously in many patients.

### 2.4 STE Secondary to Left Ventricular Hypertrophy (LVH)

**Figure 5:**
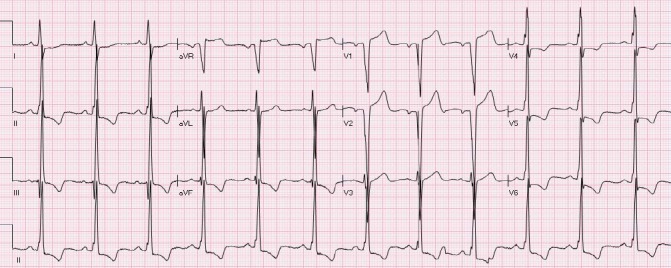
STE secondary to left ventricular hypertrophy. There are voltage criteria for LVH. There is STE in leads aVR, V1-V3 and ST depression with negative T waves in leads I, II, III, aVF, V4-V6

It is observed most commonly in leads V1 – V3 (same leads as the normal variant pattern) (Figure 5). Common associated findings include QRS voltage fulfilling criteria for LVH and a ST depression in the lateral leads I, aVL, V5 and V6. Concomitant ST elevation in AVR may also be seen. It is of utmost importance that it be differentiated from left main territory ischemia pattern (STE in AVR and V1, ST depression in precordial and inferior leads) It has to be kept in mind that patient with LBBB and LVH do not have the usual cutoffs for STE (Thygesen, et al., 2007). Moreover, LVH may present in combination with early repolarization or intraventricular conduction delay and more than one pattern of NISTE may be seen in the same patient.

### 2.5 Acute Pericarditis

**Figure 6:**
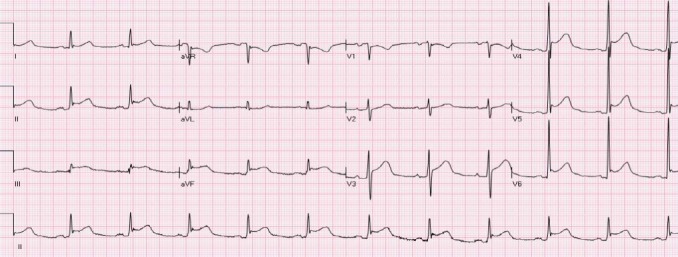
Diffuse STE in leads I, II, III, aVF, V2-V6 with ST depression in leads aVR and V1, compatible with acute pericarditis

Diffuse STE with reciprocal ST depressions in leads AVR and V1 are hallmark features of stage 1 pericarditis (Figure 6). Diffuse nature of STE differentiates it from STEMI due to an occlusion of a single coronary artery. Stage 2 pericarditis may involve PR depressions. However, after acute STEMI or cardiac surgery, focal pericarditis may lead to localized STE with ST depression in leads other than AVR and V1 and can be confused with a STEMI (Ariyarajah & Spodick, 2007).

### 2.6 STE Secondary to IVCD

**Figure 7:**
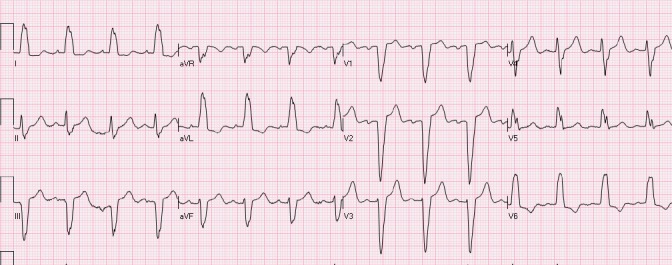
STE secondary to LBBB. There is discordant STE in leads II, III and aVF and V1-V3 and ST depression in leads I, aVL and V6

LBBB is associated with secondary ST- T wave abnormality which leads to deviation of ST segment in the opposite direction to major QRS deflection (Figure 7). It is in contrast to primary ST- T wave changes seen with STEMI which manifests as ST deviation concordant with QRS complex deflection. Given the secondary nature of ST- T changes in LBBB, STE is seen in lead V1- V3, with negative QRS deflection in these leads. Sgarbossa’s criteria (Sgarbossa, 2000; Sgarbossa et al., 1996) serve as a tool to diagnose STEMI in patients with baseline LBBB. These criteria are: STE > 0.1 mV concordant with QRS deflection, ST depression > 0.1mV in leads V1, V2 and V3 or STE of > 0.5 mV discordant with QRS complex. Diagnostic ability of Sgarbossa’s criteria was studied and validated in a prospective population based study (Al-Faleh et al., 2006). Low sensitivity (<16%) of the criteria must be kept in mind, however (Gula, Dick, & Massel, 2003; Li, Walden, Marcilla, & Gallagher, 2000). It must also be remembered that in patients with LBBB magnitude of ST deviation varies with changes in heart rate, QRS duration and with positioning of electrodes (particularly in patients with left axis deviation in anterior and lateral precordial leads).

**Figure 8:**
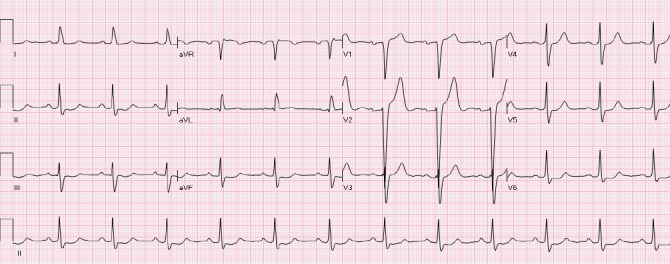
A patient with nonspecific intraventricular conduction delay (IVCD). There is marked STE in leads V1-V2 and ST depression in leads II, III, aVF and V6. There is also mild STE in lead aVR

**Figure 9:**
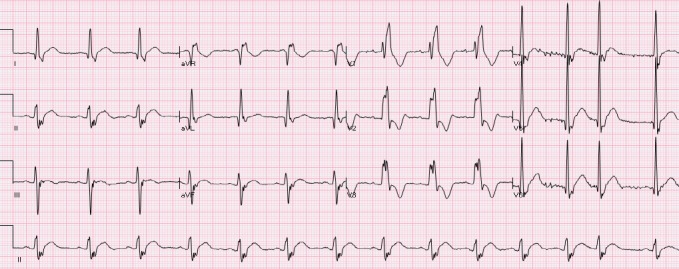
A patient with RBBB and left anterior fascicular block. There is mild STE in leads I, II, aVL, aVF and V4-V6. This patient had non ischemic dilated cardiomyopathy and his cardiac markers remained negative. This pattern is chronic and has not changed over one year

**Figure 10:**
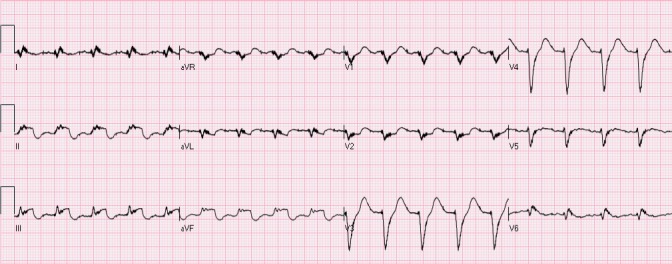
A patient with bi-ventricular pacemaker. There is STE in leads II, III, aVF and ST depression in leads aVL, V2-V4

Secondary repolarization changes, as seen with LBBB, may also be seen with non specific IVCD (Figure 8). Comparison with baseline ECG or serial observation for dynamic changes helps in accurate diagnosis. Interpretation of ST deviation is usually thought not to be affected by presence of Right bundle branch block (RBBB). However, ST depressions may be seen in leads V1- V3 in patients with RBBB and need to be differentiated from infero- lateral or posterior STEMI. Occasionally concordant STE may be seen in patients with RBBB, although generally believed to indicate ischemia, NISTE can also frequently lead to it (Figure 9). NISTE is also seen in Wolf- Parkinson- White Syndrome, amplitude depending on degree of activation through accessory pathway. A growing problem is diagnosing STEMI in patients with electronic ventricular pacing. Secondary ST-T changes are commonly seen. Interestingly, QRS configuration and the degree of ST deviation may change over time, especially in patients with bi-ventricular pacing (Figure 10). Currently, there are no published criteria as how to diagnosed STEMI in such patients.

### 2.7 Brugada Syndrome

**Figure 11:**
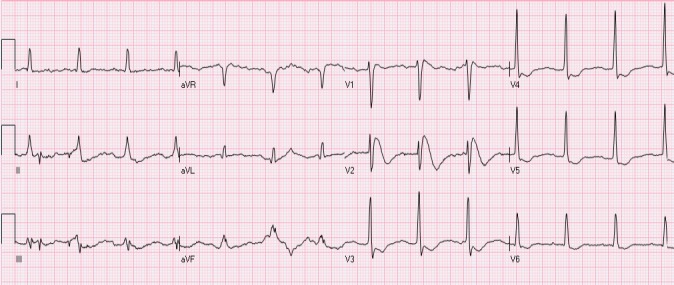
A patient with Brugada pattern. There is RsR’ pattern with STE and negative T waves in leads V1-V2

The Brugada syndrome associated NISTE manifests as RBBB-like pattern and STE in anterior leads (Figure 11) (Antzelevitch & Nof, 2008; Brugada, Brugada, & Brugada, 2000). There is a higher incidence of ventricular tachyarrhythmias and sudden cardiac death in these patients. Three types of Brugada pattern have been described. Type 1 is characterized electrocardiographically by a coved-type STE > 0.2 mV and an inverted T wave in leads V1 – V3 (right precordial leads). Other diagnostic features include documented ventricular fibrillation, polymorphic ventricular tachycardia, family history of sudden cardiac death at age < 45 years, similar ECG findings in relatives, inducibility of ventricular tachycardia with programmed electrical stimulation, agonal breathing at night and syncope (Antzelevitch et al., 2005). Type 2 has saddle back pattern with initial STE of > 0.2 mV with positive or biphasic T wave. Type 3 Brugada has a coved or saddle back STE of < 0.1 mV. Type 2 and type 3 ECG pattern are not considered diagnostic for the Brugada’s syndrome and specialized electrophysiologic testing is required. Also, the variation in ST segments in these patients over time and even days must be kept in mind (Antzelevitch, et al., 2005).

### 2.8 Takotsubo Syndrome (Apical Ballooning Syndrome)

**Figure 12:**
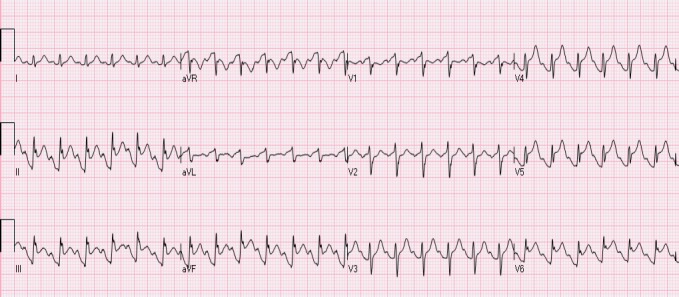
A patient with Takotsubo syndrome. There is sinus tachycardia with STE in leads II, III, aVF, V4-V6 and ST depression in leads aVR, V1. Coronary angiography showed absence of significant coronary narrowing

Takotsubo’s syndrome is a form of reversible myocardial dysfunction which is preceded by intense physical or emotional stress and is more commonly seen in post menopausal women. Presenting features may include chest pain or dyspnea and ECG shows STE in precordial leads in up to 80% of patients (Figure 12) along with mildly positive cardiac biomarkers (Gianni et al., 2006). Other ECG changes seen with Takotsubo’s include T wave abnormalities in nearly 60% and Q waves in 30%. In many cases, the clinical presentation may be very similar to anterior STEMI (Figure 2), however, evaluation by echocardiography will reveal apical ballooning and distal hypokinesis which distinguishes it from typical anterior STEMI. However, a similar ECG and echocardiographic pattern can be seen in distal occlusion of a wrapping LAD(Carrillo, Fiol, Garcia-Niebla, & Bayes de Luna, 2010). Lead AVR is seen to have greater ST segment deviation than lead V1 in Takotsubo’s and may serve as an additional distinguishing feature from acute anterior STEMI (Kosuge et al., 2010).

### 2.9 Spontaneously Reperfused STEMI

**Figure 13:**
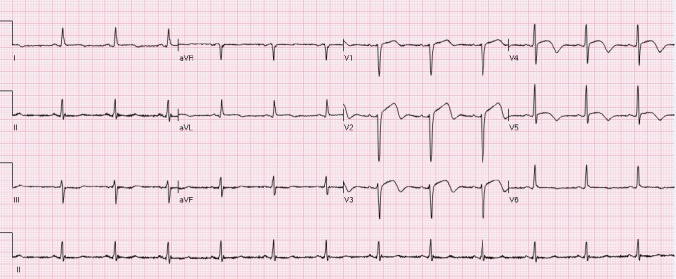
Spontaneously reperfused STEMI. There is ST elevation with terminal T wave inversion in leads v2-V5. There is also minimal ST elevation with terminal T wave inversion in leads I and aVL

The ACC/AHA STEMI guidelines recommend urgent reperfusion therapy in patients presenting within 12 hours of symptoms suggestive of myocardial ischemia and STE in ≥ 2 adjacent precordial leads (> 0.1 mV at J point) (Antman, et al., 2004). No mention is made of the need for presence of ongoing symptoms as a criterion or that of spontaneously reperfused STEMI. A significant amount of people may have (partial) resolution of symptoms by the time of presentation especially if chewable aspirin has been administered. A comparison from pre hospital ECG may show (partial) STE resolution with terminal T wave inversion. Admittedly, these patients do have a risk of reocclusion, but there is no data to support or refute the role of emergent reperfusion therapy (p-PCI or thrombolysis) in patients with spontaneously reperfused STEMI (Figure 13).

### 2.10 Left Ventricular Aneurysm

**Figure 14:**
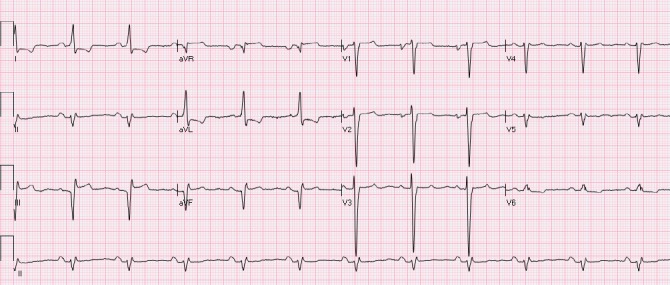
A patient with chronic STE in the inferior leads due to aneurysm. Note pathological Q waves and STE in leads III and aVF

Left Ventricular Aneurysm following a myocardial infarction may lead to persistent STE which may be indistinguishable from acute STEMI, particularly if the prior ECGs are not available. With LV aneurysm pathologic Q waves are usually seen in the same leads as those with STE. One case has been reported in which a patient was given thrombolytic therapy on 2 separate presentations as his STE were thought to be secondary to acute STEMI (http://hqmeded-ecg.blogspot.com/2008/11/65-yo-male-with-recent-rule-out.html). The magnitude of STE may change over time and is highly dependent on the heart rate, increasing with tachycardia and decreasing with slow heart rate (Figure 14).

Prompt differentiation between STEMI and NISTE may be challenging as many patients show mixed patterns of STE. It must be kept in mind that even patients with benign forms of NISTE may present with chest pain and have NSTEMI. This “pseudo” - STEMI should be differentiated from STEMI. It must be remembered that some forms of NISTE may be temporary and of varying magnitude (e.g. Brugada syndrome, early repolarization). These dynamic changes should be differentiated from evolving changes of STEMI. The ACC/AHA guidelines (Antman, et al., 2004; Antman, et al., 2008) recommend that reperfusion decisions be made within 10 minutes of initial ECG highlighting the time dependant nature of benefit from reperfusion. Quick and accurate interpretation is of necessary to reduce door-to-balloon times (Bradley et al., 2006). New approaches are being implemented to reduce the time to reperfusion. One such approach involves pre- hospital wireless transmission of ECG to on call cardiologists by Emergency Medical Services (EMS). Reduced door-to-balloon and door-to- needle time have been seen with this strategy (Dhruva et al., 2007; Garvey, MacLeod, Sopko, & Hand, 2006; Terkelsen et al., 2002). Besides interpreting the ECG, cardiologists can even communicate with the patient and EMS staff using a mobile phone (Sorensen et al., 2011). In the United States however, such strategies have not been implemented as yet. Even when ECGs are wirelessly transmitted, it is without patient details to comply with The Health Insurance Portability and Accountability Act (HIPAA). This precludes the interpreter from comparing them to previous ECGs from patients chart. Admittedly, as this may improve the sensitivity of diagnosing STEMI, its effect on the specificity of calling STEMI remains unclear. Overall, this may lead to overutilization of resources by unnecessary activation of catheterization laboratory. Jayroe et al (Jayroe et al., 2009) studied the variability in interpretation of 15 experienced electrocardiographers in telling acute STEMI needing pPCI from NISTE. Interestingly, a wide variability (7.8- 33%) was seen in recommending pPCI, but in reality only 8 patients (7%) had adjudicated STEMI. Sensitivity of interpreters ranged from 50- 100% (average 75%) and specificity ranged from 73%- 97% (average 85%). Significant differences were seen in reasons for calling NISTE among readers suggesting different criteria used by them. When a subsequent “real world” study was performed (Tran et al., 2011) to assess the ability of a group of 7 interventional cardiologists to distinguish between ischemic and nonischemic STE on ECGs taken from a STEMI-activation database, the interventional cardiologists performed similarly. In this study 48% of the 84 ECGs represented true STEMI confirmed angiographically. The rate for which readers recommended p-PCI varied widely between 33% to 75% among readers. Readers’ sensitivity ranged anywhere from 55% to 83% (average 71%) and specificity ranged from 32% to 86% (average only 63%). Overall, the findings of these reports underscore the need for more studies to delineate standardized criteria to differentiate between STEMI and NISTE in the general populations.

In clinical practice, many cases of true STEMI are easily identified. In those patients where differentiation is difficult, combining clinical presentation with interpretation of ECG by a skilled electrocardiographer who has access to previous tracings and occasionally, repeating ECG can increase the yield of interpretation.
